# Enhancing Wear Resistance and Surface Hardness of Three-Dimensionally Printed Denture Teeth Through Post-Treatment Under Elevated Temperature and Pressure

**DOI:** 10.3390/jfb17090446

**Published:** 2026-09-03

**Authors:** Shanglin Wu, Yuriko Komagamine, Bohan Tang, Tamaki Hada, Aung Soe Myint, Pong Pongprueksa, Manabu Kanazawa

**Affiliations:** 1 Department of Gerodontology and Oral Rehabilitation, Graduate School of Medical and Dental Sciences, Institute of Science Tokyo, Tokyo 113-8549, Japan; s.wu.gerd@tmd.ac.jp (S.W.); tang.bohan@tmd.ac.jp (B.T.); aung.m.10a9@m.isct.ac.jp (A.S.M.); m.kanazawa.gerd@tmd.ac.jp (M.K.); 2 Department of Oral Devices and Materials, Graduate School of Medical and Dental Sciences, Institute of Science Tokyo, Tokyo 152-8550, Japan; t.hada.gerd@tmd.ac.jp; 3 Department of Operative Dentistry and Endodontics, Faculty of Dentistry, Mahidol University, Bangkok 10400, Thailand; pong.pon@mahidol.ac.th

**Keywords:** post-treatment, post-polymerization, wear resistance, Vickers hardness, 3D-printed denture teeth resins

## Abstract

Three-dimensionally (3D)-printed denture teeth are increasingly used in clinical practice; however, their wear resistance and surface hardness may be insufficient for long-term clinical application. Moreover, the effect of post-treatment under elevated temperature and pressure on these properties remains unclear. This study aimed to evaluate the effect of such post-treatment on the wear resistance and Vickers hardness of 3D-printed denture teeth resin. One hundred disk-shaped specimens (10 × 2 mm) were 3D-printed using Dima denture teeth resin and randomly assigned to four post-treatment groups (121 °C at 2 bar for 4, 30, 60, and 90 min) and one untreated control group. Within each group, specimens were allocated for wear resistance (*n* = 10) and Vickers hardness (*n* = 10) testing. Wear resistance was measured using an impact-sliding wear testing machine in a polymethyl methacrylate slurry, while Vickers hardness was measured using a hardness tester under a 50 g load with a 15 s dwell time. Statistical analyses included one-way analysis of variance (ANOVA), Kruskal–Wallis, and Welch’s ANOVA (α = 0.05). All post-treatment groups exhibited significantly lower depth loss and higher hardness than the control group (*p* < 0.05). Specimens post-treated for ≥30 min exhibited significantly lower volume loss than the control group (*p* < 0.05). The 121 °C/90 min group demonstrated the highest wear resistance and Vickers hardness. Within the limitations of this study, post-treatment under elevated temperature and pressure significantly improved the wear resistance and Vickers hardness of the tested 3D-printed denture teeth resin, with the 90 min protocol yielding the greatest enhancement. Thus, thermal-pressure post-treatment may be a promising approach for enhancing the mechanical performance of additively manufactured denture teeth.

## 1. Introduction

Computer-aided design and computer-aided manufacturing (CAD–CAM) technologies have emerged as efficient alternatives to conventional techniques for the fabrication of complete dentures [1,2,3]. Digital dentures are fabricated using subtractive manufacturing (SM) or additive manufacturing (AM); SM removes material from a pre-polymerized polymethyl methacrylate (PMMA) block, whereas AM forms prostheses in a layer-by-layer manner [4,5]. Digital light processing (DLP) is widely adopted among AM technologies owing to its high efficiency [6,7].

Despite these advantages, 3D-printed denture teeth generally exhibit inferior wear resistance and surface hardness compared with those of their milled counterparts [8,9,10,11]. Previous studies have consistently reported lower Vickers hardness values for 3D-printed denture resins than for milled denture resins [10,11]. Similarly, Shin et al. reported that Dima denture teeth resin demonstrated the greatest wear among the evaluated 3D-printed materials. These mechanical limitations are attributed to incomplete polymerization, interlayer interfaces, and residual monomers inherent to layer-by-layer fabrication [12]. Excessive wear may result in the loss of vertical dimension of occlusion, reduced masticatory efficiency, muscle fatigue, temporomandibular joint discomfort, and compromised denture stability and retention, ultimately affecting oral function and quality of life [13,14,15,16]. These limitations highlight the need for additional post-treatment strategies to improve long-term performance.

Post-treatment methods have been proposed to enhance the degree of conversion and mechanical properties of AM resins. For instance, heat treatment in water at 100 °C significantly improved the degree of conversion (DC) and cytocompatibility of NextDent C&B resin [17]; moreover, autoclave post-treatment at 132 °C reduced residual monomer elution and increased the surface microhardness of stereolithography-fabricated acrylic resin [18]. These improvements are largely attributed to increased molecular mobility at temperatures approaching or exceeding the glass transition temperature, enabling further polymer chain rearrangement and crosslinking [17,18]. However, the effect of elevated temperature and pressure on the wear resistance of DLP-printed denture teeth remains unexplored.

Therefore, this study aimed to investigate the effect of post-treatment under elevated temperature and pressure on the three-body wear resistance and Vickers hardness of 3D-printed denture teeth resin. The null hypothesis stated that post-treatment would exert no effect on the wear resistance or Vickers hardness of 3D-printed denture teeth resin. Given that this investigation was limited to a single commercially available resin material (Dima), the results should be interpreted within this scope. Further studies are warranted to confirm whether similar effects are observed across different resin systems.

## 2. Materials and Methods

A methyl methacrylate-based denture teeth resin (Dima Print Denture Teeth A3; Kulzer GmbH, Hanau, Germany) was used in this study. According to the manufacturer’s safety data sheet, the resin is a light-curable mixture comprising methacrylate monomer (40–60%), urethane methacrylate (15–45%), propylidynetrimethyl trimethacrylate (1–10%), photoinitiator (≤3%), inhibitor (≤1%), and pigments (≤2.5% each). The material contains approximately 40 wt% inorganic filler. The selection of this specific resin was based on several considerations. First, Dima represents one of the limited commercially available materials that are specifically indicated for the 3D printing of denture teeth, thereby rendering it clinically relevant for the intended application. Second, a previous study on the wear behavior of Dima teeth resin has demonstrated that it exhibits substantially greater wear than other additively manufactured materials, which underscores the need for enhancing its wear resistance through appropriate post-treatment protocols [12]. Collectively, these factors supported the selection of Dima resin as the test material for the present study.

Based on a power analysis using G*Power software (version 3.1.9.7; Heinrich-Heine University, Düsseldorf, Germany), a sample size of 50 specimens per test method, comprising 10 specimens per group, was required to achieve 80% power at a 5% alpha level. Therefore, 100 disk-shaped specimens were fabricated with a diameter of 10 mm and a thickness of 2 mm: 50 specimens for wear testing and 50 specimens for Vickers hardness testing. These dimensions were selected to standardize the specimen geometry for wear and hardness testing and are consistent with those of previous studies on dental resin materials [19]. Specimens were modeled using CAD software (Geomagic Freeform, version 2021; 3D Systems, Rock Hill, SC, USA), and the designs were exported as stereolithography files. The disk-shaped specimens were printed without a support structure at a 0° angle, with a layer thickness of 50 µm, using a DLP printer (Cara Print 4.0; Kulzer GmbH, Hanau, Germany) equipped with 385 nm light-emitting diodes. A 50 µm layer thickness was selected, as previous studies have demonstrated superior mechanical properties with this thickness compared to thicker layers owing to enhanced interlayer polymerization and reduced internal defects [20]. Upon completion of printing, the samples were cleaned with 99% isopropyl alcohol using a cleaning machine (Cara Print Clean; Kulzer GmbH, Hanau, Germany). After air-drying, the specimens were placed in a glycerol bath and post-cured for 10 min on both sides using a light-curing unit (HiLite Power 3D; Kulzer GmbH, Hanau, Germany) with a wavelength range of 390–540 nm at 200 W. Specimens were then placed in an ultrasonic bath for washing with distilled water to remove residual glycerol.

The finished specimens were grouped according to post-treatment conditions: Group I (control) specimens were stored in a dry, dark container at 23 ± 2 °C without further post-treatment. Specimens in Groups II–V underwent post-treatment at 121 °C under approximately 2 bar of pressure for 4, 30, 60, and 90 min, respectively. Pressure was generated in a sealed pressure pot (TAF-SR-300; TAIATSU Corporation, Kanagawa, Japan) containing a small amount of tap water, which produced pressurized saturated steam during heating in a drying oven (OFW-300V; AS ONE Corporation, Osaka, Japan). Accordingly, the post-treatment environment resembled a steam autoclave, in which both elevated temperature and steam pressure were applied simultaneously. However, oxygen levels were not precisely controlled owing to the residual air within the chamber, which is acknowledged as a limitation of the present study. After post-treatment, all specimens were stored at room temperature (23 ± 2 °C) in a dry, dark environment for 24 h.

Of the 100 disk-shaped specimens, 50 were allocated to wear testing and embedded in auto-polymerizing acrylic resin (Unifast, GC Corporation, Tokyo, Japan) at the center of clear acrylic rings. The remaining 50 specimens were used for Vickers hardness testing. All specimens were dried at 37 °C for 24 h. For wear testing, specimen surfaces were manually ground and polished with wet silicon carbide papers of 800, 1500, and 4000 grit (Sankyo Rikagaku Corporation, Izumi, Japan), with 30 s of polishing per grit. This protocol was adopted based on previous evidence that laboratory polishing up to 4000 grit produces sufficiently smooth surfaces for minimizing roughness-related variability in wear assessment [21]. An impact-sliding wear testing (ISWT) machine (K655-05; Tokyo Giken, Tokyo, Japan) was used to perform the wear test. The ISWT methodology has been established and validated in previous dental wear studies [12]. A stainless-steel rod with a convex hemispherical tip (diameter: 4 mm) was positioned on the upper holder of the wear testing machine to align with the center of the abrasion specimen. This dimension was selected based on the following considerations. First, the 2 mm radius approximates the physiological curvature of natural posterior cusps, which reportedly range from 0.6 to 2.5 mm [22]. Second, standardized hemispherical antagonists are routinely employed in dental wear testing to minimize geometric variability of the opposing surface. This methodological approach ensures that the measured wear behavior predominantly reflects the intrinsic properties of the test material rather than variations in antagonist morphology, as supported by previous in vitro investigations utilizing hemispherical specimens [12].

The specimens, embedded in acrylic rings, were fixed at the bottom of the water-filled tank of the ISWT machine, which was set to generate simultaneous vertical and horizontal movements under thermomechanical loading at 37 °C. The impact sliding wear test was conducted under distilled water at 37 °C to simulate the oral environment, as the lubricating effect of saliva is known to significantly influence the wear behavior of dental materials. The presence of water may also reduce frictional heat buildup and provide more clinically relevant wear conditions compared with dry testing. This approach is consistent with the established ISWT methodology and other in vitro wear studies that have employed distilled water as a lubricant [12]. It should be noted that the 37 °C test temperature serves a different purpose from thermocycling protocols (5 °C and 55 °C). The 37 °C condition was adopted to simulate intraoral temperature during function, whereas thermocycling is designed to evaluate thermal aging effects. These two conditions address different research questions and are therefore not directly comparable. A vertical load of 49 N was applied during each chewing cycle, comprising a 5 mm vertical motion and a 2 mm horizontal motion, as described previously [23]. This load falls within the range of normal masticatory forces, as normal chewing forces in natural dentition typically range from 10 to 50 N [24], and represents the upper end of this physiological range. Consistent with this, the 49 N load has been widely employed in previous in vitro wear studies of dental materials [25].

**Figure 1:**
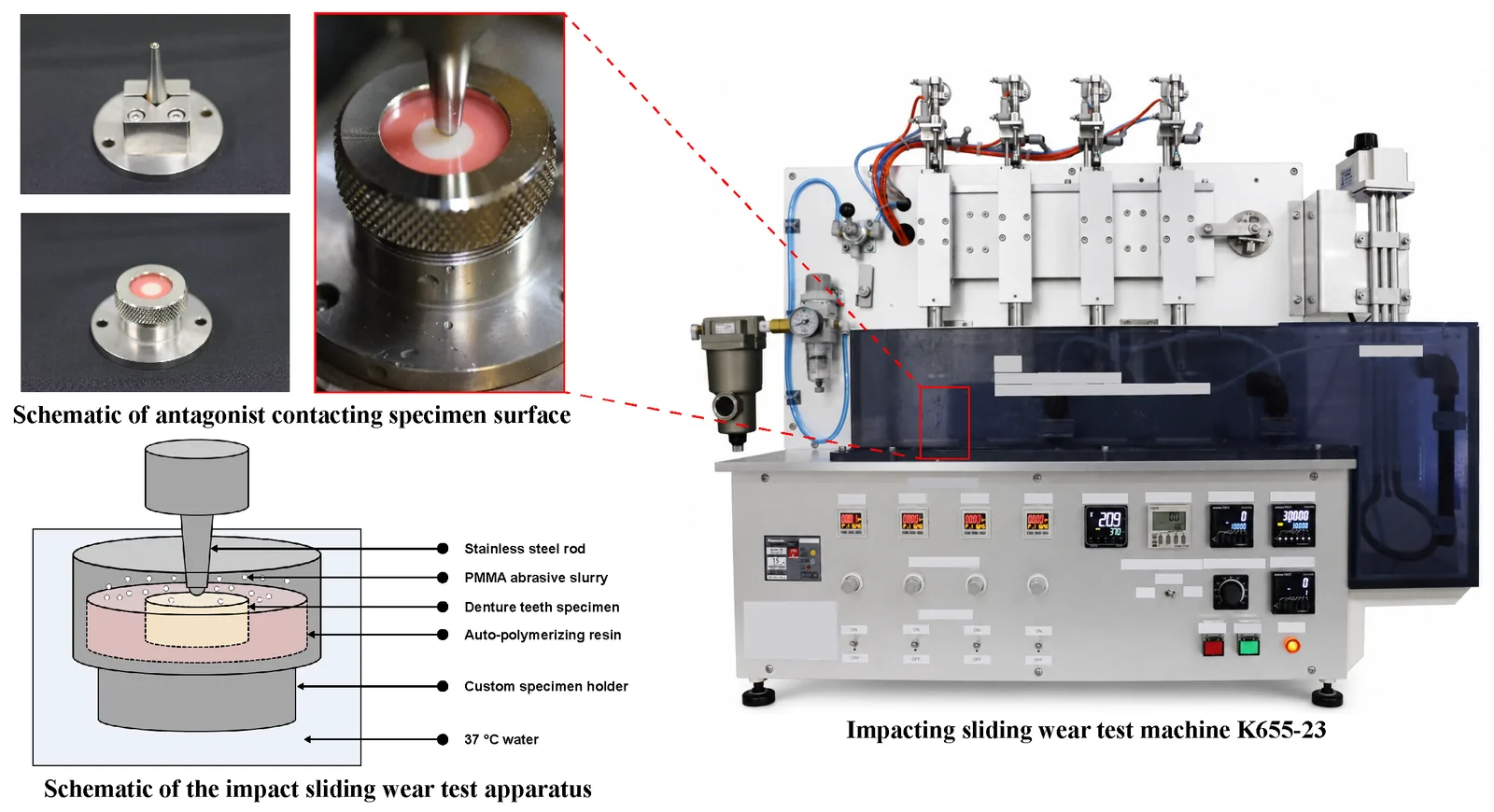
Impact-sliding wear testing apparatus. PMMA, polymethyl methacrylate.

A total of 50,000 cycles were completed at a frequency of 1 Hz [26]. The entire impact sliding test was conducted below the water level, with heating and evaporation monitored at 37 °C. The specimens were covered with a slurry of PMMA powder (Techpolymer MBX-50; Sekisui Plastics Co., Ltd., Tokyo, Japan) mixed with tap water at a 1:1 weight ratio to simulate a food bolus during the wear test [12]. The slurry was removed and renewed every 5000 cycles (Figure 1).

**Figure 2:**
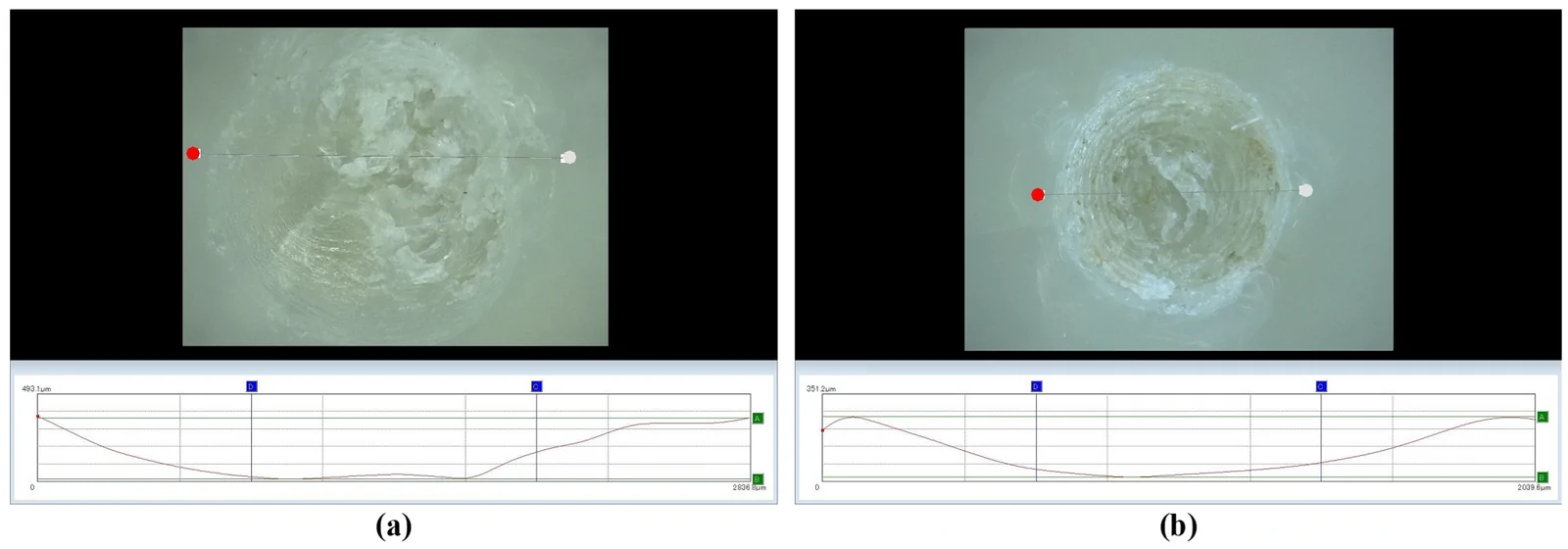
Representative digital microscope images (100× magnification) of specimens tested after 50,000 loading cycles: (**a**) control group, (**b**) 121 °C/90 min group.

After completing 50,000 loading cycles, the specimens were rinsed under running water and dried. The maximal depth loss and wear volume loss of each specimen were measured using a digital charge-coupled device microscope (VHX1000; KEYENCE Corporation, Osaka, Japan) at 100× magnification with 5 µm intervals along the *z*-axis. Reference planes of the specimens were horizontally aligned by defining three non-collinear points on the unabraded area adjacent to the reference markings [26]. Three measurements were taken for each specimen, and the mean values were calculated. Group means and standard deviations (SDs) for all 50 specimens were recorded (Figure 2).

Specimens for wear testing were polished up to 4000 grit, whereas those for hardness testing were polished up to 1500 grit. This discrepancy in polishing protocols was adopted because wear testing requires a smoother surface finish to minimize roughness-related variability in wear assessment [21], whereas hardness measurements are less sensitive to surface finish beyond 1500 grit, as previously established by Campanha et al. [27] for Vickers hardness testing of denture teeth resins.

For hardness testing, 50 disk-shaped specimens were sequentially polished with wet silicon carbide papers of 800, 1000, and 1500 grit (Sankyo Rikagaku Corporation, Izumi, Japan), each for 30 s, in accordance with the methodology of Campanha et al. [27], who employed 1500-grit silicon carbide paper for surface preparation prior to Vickers hardness testing of denture teeth resins. The hardness was measured using a Vickers hardness tester (MVK-H2; Akashi Corporation, Kanagawa, Japan) under a 50 g indentation force with a 15 s dwell time. This load was consistent with that used in previous studies on resin composite materials [28]. Each specimen was measured at five predefined locations uniformly distributed across the polished surface, avoiding the specimen edges and previous indentation sites [29]. Mean values were calculated, and group averages with SDs for all 50 specimens were recorded.

All data were analyzed using SPSS software (version 23; IBM; Armonk, NY, USA). Data distribution was assessed using the Shapiro–Wilk test, and homogeneity of variances was evaluated using Levene’s test to determine the appropriate statistical methods. For maximal depth loss, the Shapiro–Wilk test confirmed normal distribution across all groups (*p* > 0.05), and Levene’s test indicated homogeneity of variances (F = 2.527, *p* = 0.058); therefore, one-way ANOVA followed by Tukey’s HSD post hoc test was employed. For volume loss, the Shapiro–Wilk test revealed non-normal distribution in groups (*p* = 0.008, *p* = 0.038), and Levene’s test confirmed unequal variances across groups (F = 37.682, *p* < 0.001); therefore, the non-parametric Kruskal–Wallis test was used, with post hoc comparisons performed using the Mann–Whitney U test with Bonferroni correction (α = 0.05). For Vickers hardness, although the Shapiro–Wilk test confirmed normal distribution across all groups (*p* > 0.05), Levene’s test indicated unequal variances (F = 6.595, *p* < 0.001); therefore, Welch’s ANOVA followed by Dunnett’s T3 post hoc test was employed. The relationship between maximal depth loss and wear volume loss was assessed using Spearman’s rank correlation coefficient due to the non-normal distribution of volumetric loss (α = 0.05).

## 3. Results

**Figure 3:**
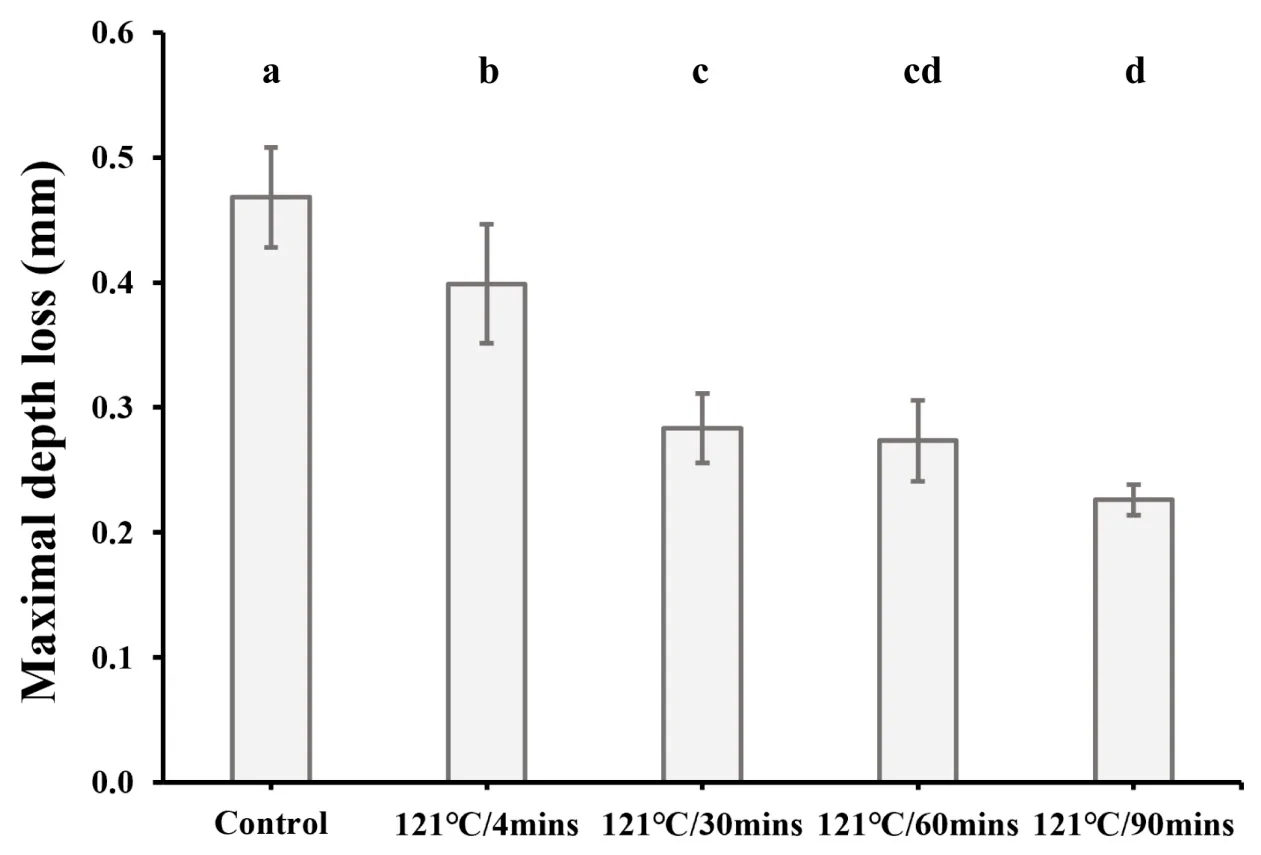
Maximal depth loss according to post-treatment conditions. Data are presented as mean ± standard deviation (SD). Different lowercase letters indicate statistically significant differences across groups (*p* < 0.05, *n* = 50).

**Figure 4:**
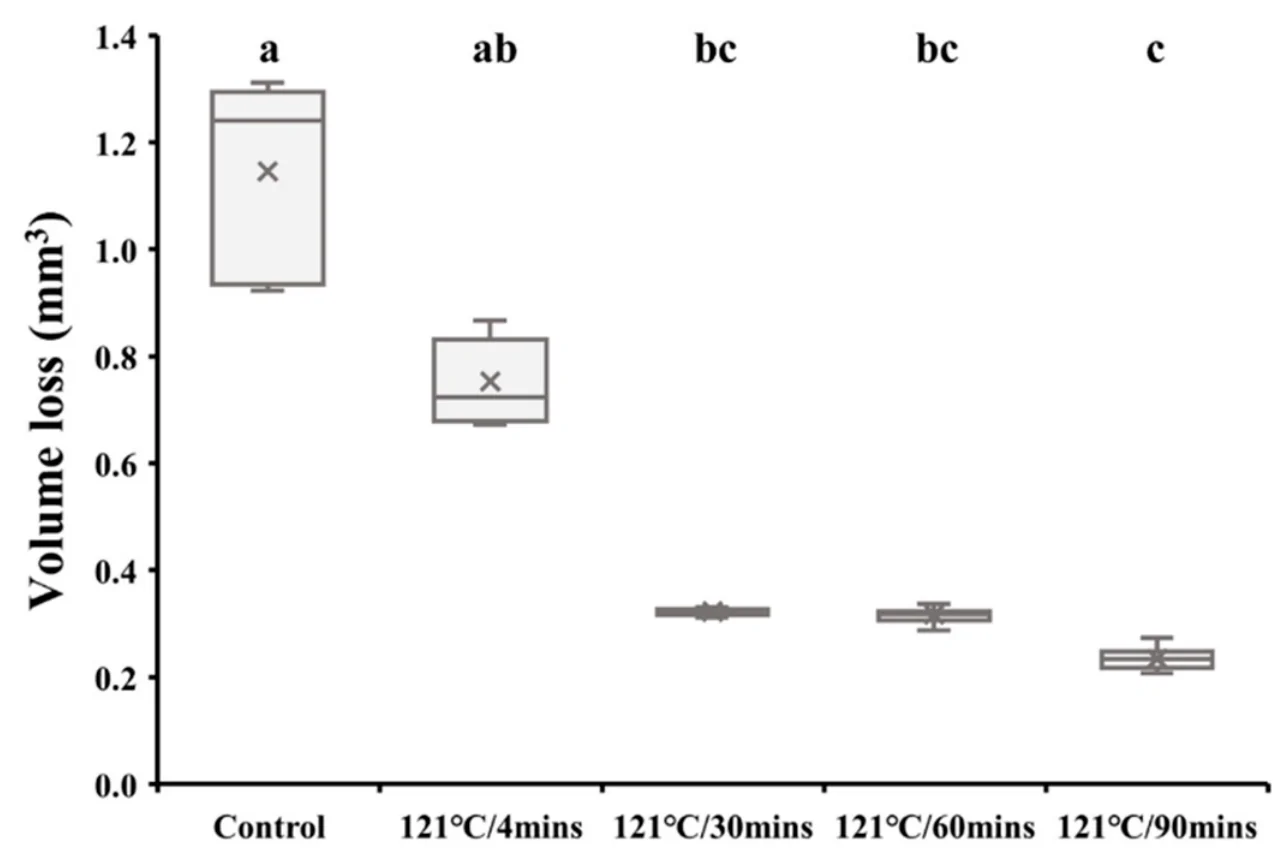
Volume loss according to post-treatment conditions. Data are presented as median ± interquartile range (IQR). Different lowercase letters indicate statistically significant differences across groups (*p* < 0.05, *n* = 50).

**Figure 5:**
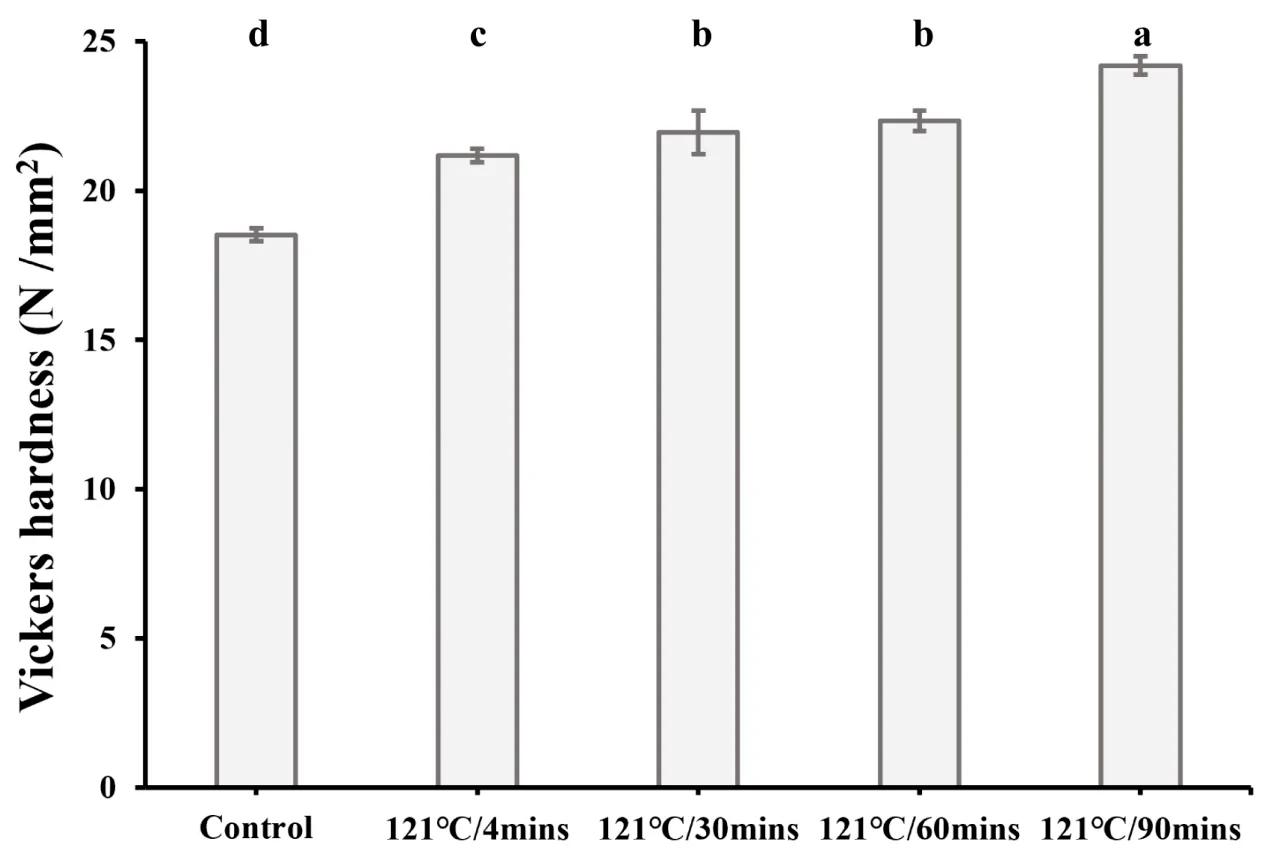
Vickers hardness according to post-treatment conditions. Data are presented as mean ± standard deviation (SD). Different lowercase letters indicate statistically significant differences across groups (*p* < 0.05, *n* = 50).

Figure 3, Figure 4 and Figure 5 present the descriptive statistics for maximal depth loss, volume loss, and surface hardness. All three parameters demonstrated statistically significant differences among the post-treatment groups (*p* < 0.001). Maximal depth loss decreased with increasing post-treatment duration (*p* < 0.001). The control group exhibited the highest maximal depth loss (0.47 ± 0.06 mm), whereas the 121 °C/90 min group exhibited the lowest (0.23 ± 0.02 mm). All post-treatment groups demonstrated significantly lower maximal depth loss than did the control group (*p* < 0.001, η^2^ = 0.913). The greatest volume loss occurred in the control group (median 1.15 mm^3^, IQR 0.93−1.29 mm^3^), whereas the least occurred in the 121 °C/90 min group (median 0.23 mm^3^, IQR 0.22–0.25 mm^3^). The 121 °C/30-, 60-, and 90 min groups demonstrated significantly lower volume loss than the control group (*p* < 0.001, 0.72 < r < 0.89). A strong positive correlation was identified between the maximal depth and wear volume losses (Spearman’s ρ = 0.97, *p* < 0.001), indicating a strong association between greater depth loss and greater overall material loss. The highest hardness value was observed in the 121 °C/90 min group (24.19 ± 0.31), whereas the lowest value was observed in the control group (18.52 ± 0.22). All post-treatment groups demonstrated significantly higher hardness values than the control group (*p* < 0.001, ω^2^ = 0.971).

## 4. Discussion

The wear resistance and Vickers hardness of the DLP-fabricated denture teeth resin evaluated in this study varied significantly according to the post-treatment duration. Therefore, the null hypothesis was rejected.

Wear resistance is essential for long-term functional efficiency and esthetic stability of denture teeth [30]. In this study, post-treatment under elevated temperature and pressure significantly improved the wear resistance of the tested 3D-printed resin. The maximal depth and volume losses demonstrated a significant decreasing trend with increasing post-treatment duration. These findings align with those of previous studies by Kim et al. [31] and Wendt et al. [32], which demonstrated that heat-based polymerization enhances the mechanical properties and wear resistance of dental resins. A strong positive correlation was observed between maximal depth and wear volume losses (Spearman’s ρ = 0.97, *p* < 0.001). This finding suggests that depth measurements may serve as a reliable indicator of volume loss of the tested material, potentially simplifying future screening methods, which may reduce the need for complex 3D volumetric analyses. All post-treatment groups demonstrated significantly higher hardness than that of the control group, which likely contributed to reduced depth and volume loss through improved resistance to plastic deformation and abrasion, indicating enhanced mechanical performance under simulated functional loading. These findings align with those of previous studies showing that extended post-curing durations and higher curing temperatures improve the mechanical properties of additively manufactured resins [33,34].

The improvements observed in this study may be attributed to several mechanisms. Elevated temperature increases free-radical mobility, facilitating further polymerization and crosslinking within the resin matrix, thereby enhancing mechanical strength and surface stability [35]. Pressure application may reduce intermolecular distances and decrease free volume, resulting in a denser, more highly crosslinked polymer network [36], and may further contribute to the collapse of microscopic voids or porosities inherent to the layer-by-layer AM process, thereby improving structural integrity and wear resistance [37]. Combined thermal- and pressure-assisted post-treatment may also promote stress homogenization within the resin matrix, reducing internal stress concentrations that might otherwise accelerate wear [38,39].

Critically, these mechanistic interpretations are not merely speculative. A previous study evaluating identical Dima denture teeth resin under the same post-treatment conditions (121 °C/90 min) reported a substantial increase in the degree of conversion (DC) from 63.42% to 88.16% [40], providing direct published evidence that thermal post-treatment indeed enhances polymerization within this specific material system. This DC improvement correlates well with the enhanced mechanical properties observed in our study, supporting the proposed mechanism of post-curing-driven network densification. However, it must be explicitly acknowledged that while this DC evidence is derived from published data on the same material, crosslink density, free volume reduction, and stress homogenization were not directly measured or verified in the present study. Therefore, these inferred mechanisms, though strongly supported by the published literature, should be interpreted with caution. Future studies employing direct characterization techniques (crosslink density analysis, positron annihilation spectroscopy for free volume, or residual stress measurements) are warranted to conclusively validate these mechanistic pathways.

Although the mechanical properties evaluated in this study improved significantly, post-treatment may raise concerns regarding the dimensional stability of additively manufactured components, as thermal expansion, stress relaxation, or internal structural rearrangement could potentially cause deformation [41]. Our previous study found that the 121 °C/90 min group exhibited the highest error rates: length (L) 0.11% (0.08–0.11%), width (W) 0.86% (0.76–1.05%), and thickness (T) 0.47% (0.41–0.70%) [40], which are all within the acceptable threshold for polymer denture teeth (±2%) according to ISO 22112:2017 [42]. The relatively stable performance of Dima resin at 121 °C may be attributed to its highly crosslinked polymer network and optimized filler–matrix interactions, which likely enhance resistance to thermal deformation. Nevertheless, linear measurements alone may inadequately capture the complexity of dimensional alterations, warranting future three-dimensional geometric analysis to more comprehensively validate clinical applicability.

Similarly, it is unclear whether these mechanical gains reflect clinically acceptable performance or merely represent enhancement over a poor baseline, given that 3D-printed denture teeth are generally inferior to milled alternatives. To assess whether the post-treatment applied here narrows this performance gap, the values achieved in this study were compared with previously reported data for milled denture teeth. Milled PMMA denture teeth typically exhibit Vickers hardness values of approximately 20–25 N/mm^2^ [11,43,44]. In the present study, the untreated control group exhibited a Vickers hardness of 18.52 N/mm^2^, confirming the inferior baseline. Following post-treatment, the 60 min group achieved a hardness of 22.21 N/mm^2^, while the 90 min group achieved 24.19 N/mm^2^. These values fall within or exceed the range reported for milled denture teeth. Notably, Shin et al. [12] reported that Dima resin exhibited significantly higher wear volume loss and caused the greatest damage to opposing enamel compared with other additively manufactured materials (Zenith, Detax, and Veltz). Sone et al. [26] further demonstrated that DLP-fabricated denture teeth with a 0° build orientation exhibited wear volume comparable to milled specimens after 50,000 cycles, but significantly lower hardness. The present study extends these findings by demonstrating that post-treatment can improve the hardness of 3D-printed denture teeth to levels matching or exceeding those of milled alternatives, while maintaining favorable wear resistance. This suggests that combining optimized print parameters (0° build orientation) with post-treatment may effectively reduce the performance gap between additively and subtractively manufactured denture teeth, particularly when extended treatment times (≥60 min) are applied.

The selection of 121 °C and 2 bar pressure was based on both clinical relevance and material considerations, including temperature-dependent polymer chain mobility, the potential for further conversion of residual monomers, and pressure-induced network densification [18]. These conditions resemble those used in standard dental steam sterilization cycles, supporting practicality and accessibility in dental laboratory settings. In this study, the small amount of tap water placed inside the sealed pressure pot contributed to a humid, pressurized environment during heating. Although complete oxygen elimination was not achieved owing to the residual air within the chamber, the water vapor may have diluted the oxygen concentration and enhanced heat transfer efficiency [45,46].

Several methodological parameters in this study warrant consideration. Post-treatment using a steam autoclave was selected because it represents a standardized, clinically accessible protocol widely used in dental practice, allowing the precise control of temperature and pressure to ensure reproducible experimental conditions. In addition, printing parameters were standardized (50 µm layer thickness, 0° orientation) to minimize variability. A 0° build orientation has been recommended for DLP-fabricated denture teeth because it promotes uniform polymerization and reduces interlayer weakness, thereby improving mechanical properties [26]. The applied chewing force and 50,000 wear cycles were selected to simulate short-term clinical service conditions rather than long-term performance [47,48]. The 50,000-cycle simulation corresponds to approximately 2–3 months of clinical function under normal masticatory load, assuming an average of 500–1000 chewing cycles per day [48]. PMMA slurry served as the third-body medium due to water resistance and capacity to limit frictional heat buildup [49], whereas a 4 mm stainless-steel antagonist simulated occlusal contact under clinically relevant wear conditions [50]. Accordingly, extrapolation of the present findings to long-term clinical outcomes should be made with caution.

This study had certain limitations. First, the use of disk-shaped specimens rather than anatomically shaped teeth limits clinical translatability, and the evaluation of only one material means the findings are material-specific. Second, the 50,000-cycle simulation represents only 2–3 months of clinical service, and the post-treatment protocol differed from the manufacturer’s recommended method. Third, the use of different polishing protocols for wear and hardness testing, as detailed in the methods section, may affect the comparability of surface conditions between the two tests and is acknowledged as a limitation. Fourth, in the present study, post-treatment was performed before bonding to the denture base. However, the timing of post-treatment—whether before or after bonding—requires careful evaluation, as heat and pressure applied to fully processed dentures may introduce distortion at the tooth–base interface or compromise the adhesive bond [51,52,53]. Moreover, interfacial changes during clinical procedures were not evaluated, which may also affect long-term performance. Fifth, the post-treatment may affect the color stability of the denture teeth. Wu et al. [54] reported that the 121 °C/60 min and 90 min groups exhibited higher color changes, but both remained below commonly reported clinical acceptability thresholds (ΔE < 2.25). While the 90 min treatment produced the highest mechanical properties, the incremental benefit over the 60 min treatment in terms of hardness must be weighed against the additional 30 min of processing time and associated clinical or laboratory costs. For routine clinical practice, the 60 min protocol may represent a more pragmatic choice, offering substantial improvements with a shorter processing time, whereas the 90 min protocol could be reserved for cases demanding maximum mechanical performance, such as patients with parafunctional habits or implant-supported prostheses. Furthermore, the proposed mechanisms were inferred, as DC and crosslink density were not directly measured. Finally, untreated 3D-printed specimens were employed as the control group, which is appropriate for evaluating the relative effects of post-treatment parameters on the same material. However, this design does not include conventional or milled denture teeth as external benchmarks, limiting direct comparison with clinically established alternatives. Future studies should address these limitations by including conventional and milled denture teeth as controls under identical testing conditions, together with a broader range of materials, anatomically shaped specimens, and extended wear simulations, for predicting long-term clinical performance.

## 5. Conclusions

Within the limitations of this study, post-treatment improved the wear resistance and Vickers hardness of Dima denture teeth resin, with the 90 min group showing the greatest improvement among the tested durations.

## Data Availability

The original contributions presented in the study are included in the article, further inquiries can be directed to the corresponding authors.

## Abbreviations

The following abbreviations are used in this manuscript:

3D	Three-dimensional
AM	Additive manufacturing
ANOVA	Analysis of variance
CAD	Computer-aided design
CAM	Computer-aided manufacturing
DC	Degree of conversion
DLP	Digital light processing
ISWT	Impact-sliding wear testing
PMMA	Polymethyl methacrylate
SD	Standard deviation
SM	Subtractive manufacturing
SPSS	Statistical Package for the Social Sciences
